# Mapping the UK Aesthetic Medicine Industry: Practitioner Profiles, Pricing, and Socioeconomic Gradients in Botulinum Toxin Practice

**DOI:** 10.1093/asjof/ojag006

**Published:** 2026-02-11

**Authors:** Alexander Zargaran, David Zargaran, Hamid Reza Khademi Mansour, Lara Turan, Giulia Bocciardi, Yichen Liu, Anita Golash, Kian Daneshi, Ali Pirayesh, Alex Woollard, Afshin Mosahebi

## Abstract

**Background:**

The United Kingdom aesthetic botulinum toxin industry operates with limited regulatory oversight despite being a prescription-only medication. Following recent public scrutiny, regulatory reforms are imminent, warranting comprehensive analysis to inform evidence-based policy.

**Objectives:**

The aim of the study was to characterize practitioner profiles, pricing patterns, premises types, and socioeconomic gradients in the provision of aesthetic botulinum toxin across the United Kingdom.

**Methods:**

Cross-sectional analysis was conducted of practitioners administering aesthetic botulinum toxin and fillers across the UK (January–July 2025). Data were collected from publicly available websites and social media platforms. Geographic analysis used deprivation indices to examine socioeconomic patterns. Statistical methods included descriptive analysis, χ^2^ tests, and multivariable regression modeling.

**Results:**

A total of 19,701 practitioners across 5589 clinics were identified, representing a 437% increase over 2 years. Doctors comprised 28.4% of practitioners, while the proportion of nonmedical aestheticians doubled from 12% to 24.8%. Practitioner density showed a 6.7-fold increase from the least (9.4 per 100,000) to the most deprived areas (63.2 per 100,000). Doctor representation declined from 34.4% in affluent areas to 27.0% in deprived areas, while odds of nonclinical botulinum toxin administering beauty salon exposure peaked in moderately deprived areas (odds ratio [OR] = 2.18). Specialist access (dermatologists/plastic surgeons) declined significantly in Quintiles 3 to 5 (ORs 0.70-0.77). Regression analysis identified practitioner profession as the strongest pricing determinant: doctors charged 32% to 38% more than aestheticians, dentists 28% to 33% more, and nurses 2% to 4% more (all *P* < .001). Geographic context exerted modest effects, with higher-income and denser areas associated with 1% to 5% higher prices.

**Conclusions:**

The UK aesthetic medicine market has expanded rapidly, with significant differences in practitioner qualifications and treatment settings across socioeconomic groups. These findings provide key evidence to guide upcoming regulatory reforms and highlight the need for stronger safety oversight of prescription-only aesthetic procedures.

**Level of Evidence: 5 (Therapeutic):**

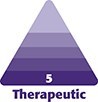

Aesthetic botulinum toxin injections are popular treatments both in the United Kingdom and internationally, with an estimated 900,000 injections carried out in the UK each year.^1^ The lack of regulation of the aesthetics industry and patient safeguards in the UK has been the subject of scrutiny and debate since the government-commissioned Keogh Review of 2013.^2^ In July 2025, the UK Health Security Agency (UKHSA) issued a public health alert for botulism following adverse reactions to aesthetic botulinum toxin procedures, citing the potential use of unlicensed products after a spike in cases presenting to hospitals across the National Health Service (NHS).^3^ A previous meta-analysis has identified the expected complication rate of facial cosmetic botulinum toxin A injections to the upper face to be ∼16%, with overall complications likely to be significantly underreported in the UK.^4,5^

In August 2025, the UK government announced plans for significant reforms to cosmetic procedures, including botulinum toxin and dermal fillers, with a proposed local authority licensing scheme, following a public consultation in 2023 for the licensing of nonsurgical cosmetic procedures in England.^6,7^ The lack of regulation and associated market growth has resulted in limited understanding of the current scale and scope of practice within the UK, precluding the effective introduction of reform. A national cohort analysis in 2023 studied 3000 websites, identifying 1224 independent clinics administering aesthetic botulinum toxin in the UK with 3667 practitioners, of whom 32% were doctors, 24% dentists, 13% nurses, and 12% nonmedical aestheticians.^8^ In 2023, there was an estimated market size of $487.4 million USD for botulinum toxin, forecast to have a compound annual growth rate of 8% from 2024 to 2030.^9^ As a prescription-only medication, botulinum toxin should be subject to greater regulatory oversight, including complication monitoring of adverse reactions by the Medicines and Healthcare Products Regulatory Agency and prohibition of advertising to the public, in line with the Advertising Standards Authority Committee of Advertising Practice code Rule 12.12; however, enforcement of this has been challenging, with a national audit identifying 88% of sampled clinics in London alone directly infringing this through advertising botulinum toxin to the public.^9^

In view of the imminent regulatory reform in the nonsurgical aesthetics industry in the UK, this study undertakes a comprehensive review of the industry, exploring practitioners’ profiles, pricing, premises, and socioeconomic differences in access; an analysis of the market is further undertaken to understand the potential implications of reform.

## METHODS

A cross-sectional analysis of clinics and practitioners was performed from January to July 2025. Since most patients consult online materials, including social media, prior to consultation for cosmetic procedures, the search strategy was designed to mirror the patient journey.^10,11^ Publicly available data were gathered from Google, Instagram, TikTok, X (formerly known as Twitter), Facebook, Fresha, and Treatwell. Searches using Boolean terms included [City] AND [Botulinum Toxin] as well as alternative brands OR [Anti Wrinkle Injection] OR [Filler]. The search was performed for the whole UK. Providers were included if they publicly advertised botulinum toxin treatments, listed at least 1 identifiable practitioner, and had a valid UK postcode; administrative-only staff were excluded. Searches were performed manually and divided among 6 independent reviewers, with overlapping cross-checks performed to ensure consistency. Data were coded for clinic name, postcode, premises type, practitioners, pricing for botulinum toxin, pricing for dermal filler injections, and practitioner professional background. Where clinics listed multiple tiers (1-, 2-, or 3-area packages), the single-area value was extracted to ensure standardization across all providers. Not all clinics publicly listed prices. No imputation was used; clinics without pricing were excluded from pricing analyses. To assess whether this could introduce bias, we compared practitioner type, region, and deprivation between clinics with and without listed prices. This comparison was performed at the practitioner level. Records for practitioner professions were cross-checked with regulatory databases, including the General Medical Council, Nursing and Midwifery Council, General Dental Council, and General Pharmaceutical Council. These registers were used solely for verification, not sampling, as they do not record whether registrants perform aesthetic injections. All data were double checked for accuracy, with only aesthetic rather than therapeutic treatments recorded.

For the analysis of socioeconomic differences, data from the Index of Multiple Deprivation (IMD) for England, the Scottish IMD, the Welsh IMD, and the Northern Ireland Multiple Deprivation Measure were used to match postcodes of clinics with deciles of deprivation.^12-15^ “Deprivation” is defined as the area-level socioeconomic disadvantage, as measured by national deprivation indices. Mean population and income data were also matched to areas using the Office for National Statistics Annual Survey for Hours and Earnings.^16^ Geographic data were identified for 91.9% of the practitioners included in the dataset.

Descriptive statistics were used to characterize the dataset and identify distribution patterns across key variables. Following a test for normality, a Kruskal–Wallis *H*-test for nonparametric data was used to assess the significance of pricing across professions and premises. Chi-square tests assessed associations between categorical variables, particularly socioeconomic deprivation and practitioner type distribution. Odds ratios (ORs) with 95% CIs were used to quantify the likelihood of encountering different practitioner types across deprivation deciles. To identify determinants of treatment pricing, ordinary least squares models were fitted with the natural log of price as the dependent variable. Outcomes included log-transformed botulinum toxin price per area (£) and dermal filler price per milliliter (£). Independent variables included practitioner profession, premises type, median household income (scaled per £10,000), and population density (scaled per 1000 persons/km^2^). Model performance was assessed using *R*^2^ and *F* statistics. All analyses were conducted using *R*. Statistical significance was set at 5%. The study utilized publicly available data and did not involve direct patient contact or identifiable personal information. All data were collected from sources intended for public access, including practitioner websites and professional directories. Findings are presented in aggregate form to protect individual privacy.

## RESULTS

Following exclusion of duplicates and removal of administrative staff, 19,701 practitioners were identified across the UK from 5589 clinics (England 79.5%, *n* = 4443; Scotland 8.3%, *n* = 462; Wales 7.2%, *n* = 405; Northern Ireland 5.0%, *n* = 279). A flowchart of the search is illustrated in Supplementary Figure 1. Doctors were the most common type of practitioner (28.4%) followed by nonmedical aestheticians (24.8%). Pricing data were found for 52% of clinics. Clinics with and without publicly listed prices had similar practitioner type (χ^2^ = 4.28, *P* = .233), premises (χ^2^ = 3.92, *P* = .270), regional (χ^2^ = 4.3, *P* = .240), and deprivation distributions (χ^2^ = 1.04, *P* = .308), with no statistically significant differences observed, indicating that missing pricing data are unlikely to distort the observed geographic or socioeconomic patterns. A full breakdown of practitioner backgrounds and mean price charged for treatments is presented in Table 1, whilst a further breakdown by country is provided in Supplementary Table 1.

**Table 1.** Professional Backgrounds of Practitioners and Mean Price Charged for Nonsurgical Aesthetic Treatments

Profession	Doctors	Nonmedical aestheticians	Nurses	Dentists	Allied health professionals
Proportion (%)	28.4	24.8	24.8	10.5	11.2
Median price for botulinum toxin (£, per area) ± IQR	187.55 ± 38.73	148.44 ± 30.09	158.32 ± 37.02	190.69 ± 19.98	157.76 ± 42.24
Median price for dermal fillers (£, per mL) ± IQR	295.18 ± 51.41	226.18 ± 36.74	254.10 ± 32.46	302.41 ± 38.76	239.75 ± 42.29

IQR, interquartile range.

Significant differences in pricing across all professions (*P* ≤ .001) were identified, with a breakdown illustrated in Table 1. Medical specialists charged the most premium pricing, with analysis identifying dermatologists and plastic surgeons as the most expensive groups for botulinum toxin (median £223.33 [$285.86 USD] and £211.01 [$270.09 USD] per area, respectively) and dermal fillers (median £334.95 [$428.74 USD] and £347.48 [$444.78 USD] per milliliter, respectively).

Analysis of premises type, as outlined in Table 2, revealed that clinics were the most common setup, followed by beauty salons. Median botulinum toxin pricing varied significantly across premises types (*H* = 42.25, *P* < 0.001), with hospitals charging the highest median prices at £170.64 ($218.42 USD) (interquartile range [IQR]: £40.00). Spas (£168.44 [$215.60 USD], IQR: £37.14) and beauty salons (£164.97 [$211.16 USD], IQR: £42.15) operated at slight discounts of 0.4% and 2.4%, respectively, compared with clinics. Mobile clinics showed the largest discount at £146.94 ($188.08 USD) (IQR: £45.40), representing a 13.1% reduction from clinic pricing. Despite statistical significance in pricing differentials by premises, the practical effect sizes were modest, with most premises types showing pricing within ±5% of the dominant clinic model, except for mobile clinics, which showed larger discounts likely reflecting lower operational overhead costs.

**Table 2.** Premises and Prices Charged

Premises type	Proportion (%)	Median botulinum toxin price (£) *±* IQR	Median dermal fillers price (£) *±* IQR
Clinic	90.7	169.08 ± 38.27	261.35 ± 54.97
Beauty salon	7.6	164.97 ± 42.15	252.52 ± 52.02
Hospital	1.2	170.64 ± 40.00	260.23 ± 48.09
Spa	0.4	168.44 ± 37.14	253.91 ± 45.93
Mobile setup	0.1	146.94 ± 45.40	243.81 ± 39.50

IQR, interquartile range.

**Figure 1.**
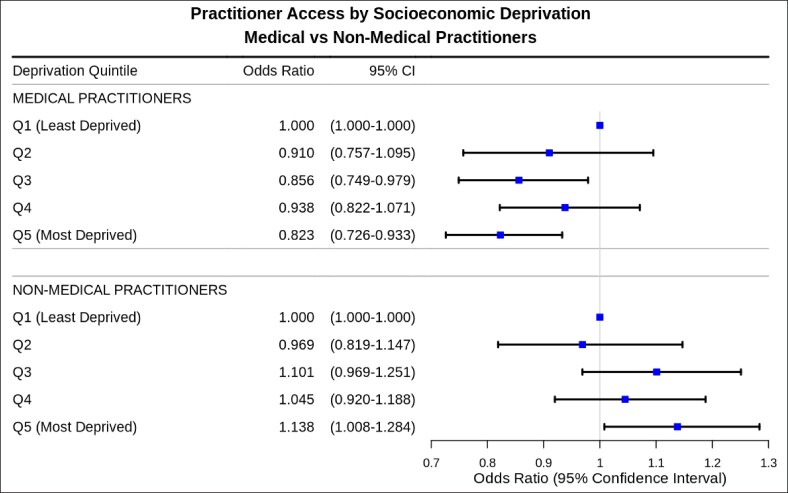
Forest plot of odds of accessing medical and nonmedical practitioners by deprivation quintile.

Chi-squared analysis revealed a significant association between socioeconomic deprivation and practitioner type distribution (χ^2^(4) = 11.40, *P* = .002). However, detailed OR analysis demonstrated that neither medical nor nonmedical practitioner access follows a systematic linear pattern across deprivation quintiles (Figure 1). Medical practitioner access showed significant reductions in Quintiles 3 and 5 compared with the least deprived quintile (Q3: OR = 0.856, 95% CI, 0.749-0.979; Q5: OR = 0.823, 95% CI, 0.726-0.933). Quintiles 2 and 4 showed nonsignificant differences from the reference category, indicating a nonlinear relationship rather than a progressive decline across the quintiles. Nonmedical practitioner access demonstrated minimal variation across most quintiles, with only the most deprived quintile showing a significant increase (Q5: OR = 1.138, 95% CI, 1.008-1.284, *P* = .036).

**Figure 2.**
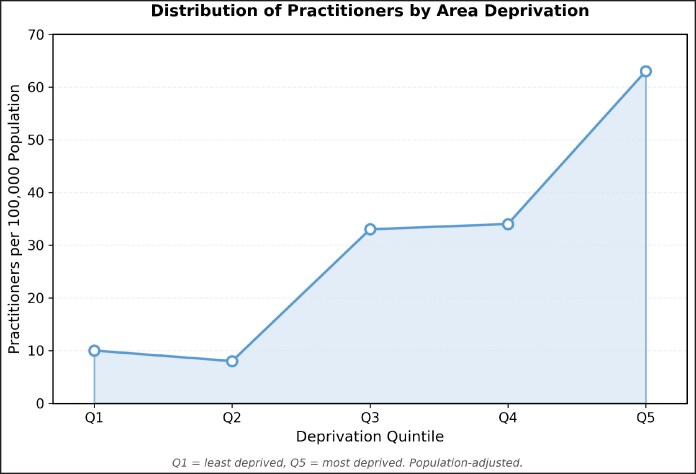
Distribution of practitioners by area deprivation.

Figure 2 outlines practitioner density stratified by IMD quintile. A strong positive correlation was observed with area deprivation, increasing from 9.4 practitioners per 100,000 population in Quintile 1 (least deprived) to 63.2 per 100,000 population in Quintile 5 (most deprived), representing a 6.7-fold difference that persisted after population adjustment (*r* = 0.932, *P* = .021)

**Figure 3.**
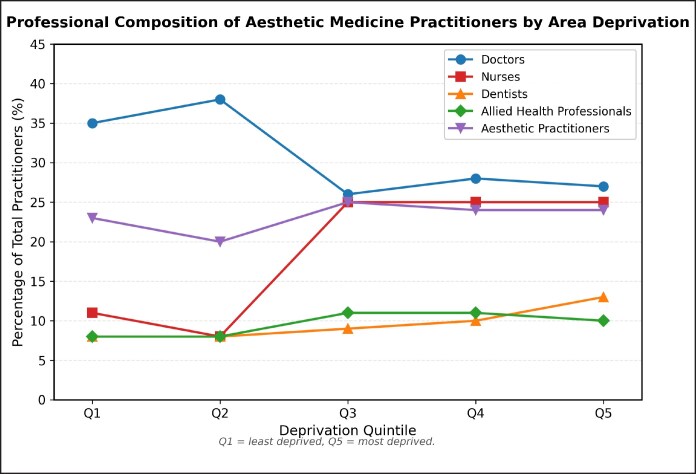
Professional composition of aesthetic medicine practitioners by area deprivation.

Professional composition varied across deprivation quintiles, as demonstrated in Figure 3; however, correlation analysis found this to be not statistically significant. Doctor representation declined from 34.4% in the least deprived areas to 27.0% in the most deprived areas, a 7.4 percentage point reduction (*r* = −0.756, *P* = .139). Conversely, allied health professionals increased from 10.6% to 12.8% across the deprivation quintiles. Nurse representation ranged from 19.8% to 25.7% across quintiles, while aestheticians comprised 22.6% to 26.8% of practitioners regardless of area deprivation level.

**Figure 4.**
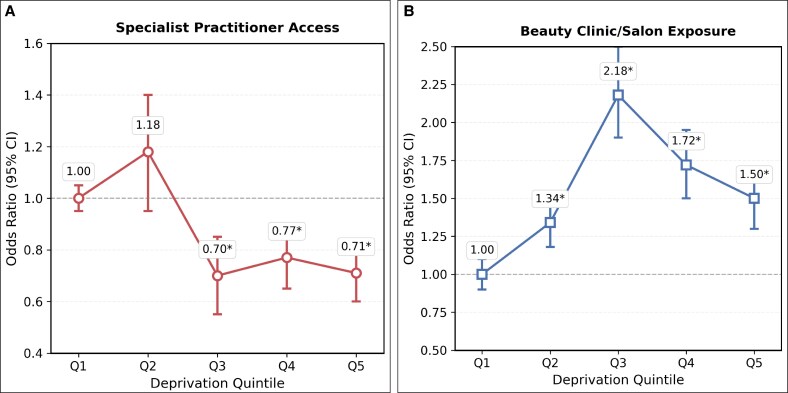
Odds of (A) specialist practitioners and (B) nonclinical beauty premises by area deprivation. *95% CI excludes 1.0 (*P* < .05). Q1 = least deprived, Q5 = most deprived.

Analysis of exposure to specialist medical treatments (dermatologists and plastic surgeons) and nonmedical premises (beauty salons and spas) also demonstrated significant differences when stratified by deprivation quintiles (Figure 4). Specialist access declined significantly from Quintile 1 to Quintiles 3 to 5, with ORs of 0.70 (*P* = .005), 0.77 (*P* < .001), and 0.71 (*P* = .009), respectively. Conversely, beauty salon exposure increased substantially with deprivation: Quintile 2 (OR = 1.34, *P* = .046), Quintile 3 (OR = 2.18, *P* < .001), Quintile 4 (OR = 1.72, *P* < .001), and Quintile 5 (OR = 1.50, *P* < .001), with peak exposure in Quintile 3 areas.

Regression analysis identified practitioner profession as the strongest determinant of pricing (Table 3). Compared with aesthetic practitioners, doctors charged 32% more for botulinum toxin and 38% more for fillers, dentists 28% and 33% more, and nurses 2% to 4% more, respectively (all *P* ≤ .001). Geographic context showed consistent but modest effects: each £10,000 increase in median income was associated with a 1% to 5% increase in prices, while each additional 1000 persons/km^2^ increased prices by 1% to 3%. Premises effects were small and inconsistent after adjustment, with hospitals charging modest discounts and other categories showing no significant variation. Overall, the models explained 43.2% of variance in botulinum toxin pricing and 50.7% of variance in filler pricing.

**Table 3.** Regression Analysis of Drivers of Botulinum Toxin and Filler Prices

Variable	Botulinum toxin (£)	Filler (£)	Botulinum toxin (%)	Filler (%)	*P*-value
Median income/£10,000	10.01	10.87	1.6	1.8	≤.001
Population density/1000/km^2^	3.26	3.54	2.1	2.3	≤.001
Hospital (vs private practice)	−2.21	−4.46	−3.3	−6.5	≤.001
Dentist (vs aesthetic practitioner)	16.24	21.89	28.3	33.0	≤.001
Doctor (vs aesthetic practitioner)	18.13	25.46	32.1	38.3	≤.001

Model performance: botulinum toxin (%) Model R2: 0.432; filler (%) Model R2: 0.507. *F* statistic >1300.

## DISCUSSION

This study provides a comprehensive cross-sectional analysis of 19,701 aesthetic medicine practitioners working across 5589 clinics in the UK. Compared with the most recent national analysis conducted in 2023 (3667 practitioners across 1224 clinics), our findings represent a 437% increase in the number of practitioners and a 357% increase in the number of clinics.^8^ This difference may not be a purely market-driven real-world proliferation and may also in part be attributable to the different methodologies employed, with the present study including social media searches. Social media has been identified as an important source for patients considering cosmetic surgery and seeking information in recent years and represents an important avenue for regulators to consider.^17,18^ However, proliferation of aesthetic practice is another explanation, with industry estimates highlighting the current and future expected growth of the area within the UK and internationally.^19^ This study further highlights the changing demographic of aesthetic practitioners. Whilst medical doctors represent the majority of practitioners, accounting for 28.4%, nearly one quarter of practitioners (24.8%) are nonmedical aestheticians compared with 12% found in the previous analysis.^8^ The proliferation of practitioners of aesthetic botulinum toxin, a prescription-only medication, who do not have professional healthcare backgrounds raises questions regarding the adequacy of training standards and highlights challenges for the impending government reforms. These challenges are not limited to practitioners, with this analysis further exploring premises. Our findings demonstrate that whilst over 90% of practice occurs in hospitals or clinics, there are a range of nonclinical settings where botulinum toxin is being administered, including beauty salons, spas, and even mobile setups, which might lack the adequate safety infrastructure for routine practice.

Analysis of socioeconomic disparities with stratification by deprivation quintile highlighted increased presence of practitioners in areas with greater deprivation, with associated lower prices. Absolute practitioner distribution remained consistent across quintiles, though greater odds were found of accessing nonmedical practitioners and lower odds of accessing medical practitioners in the most deprived areas. This was further supported by the finding of significantly lower odds of accessing specialist (dermatologists or plastic surgeons) practitioners in Quintiles 3 to 5 when compared with Quintile 1. This elucidates a pattern of greater access to more affordable aesthetic treatments administered by less specialist practitioners in the most deprived areas when compared with the least deprived areas. Our findings differ from Kim and Kang's 2024 analysis on the highest and lowest 5% income in the United States of America, who found that cosmetic treatments (both surgical and nonsurgical) were more accessible and more prevalent among the top 5%.^20^ The different findings might be attributable to differences in healthcare behaviors between the 2 countries, as well as differences in regulation, with the 2024 analysis focusing on both surgical and nonsurgical procedures using data from the American Society of Plastic Surgeons, whereas the present study explores an area with a profound lack of regulation in aesthetic botulinum toxin in the UK.

In their 2002 analysis, Alsarraf et al identified a significant association between increased prices for cosmetic facial plastic surgery and demand, positioning it as a luxury service in the marketplace.^21^ However, it is worth noting that the 2002 analysis, which took place in the United States is unlikely to have included aesthetic botulinum toxin, which only received FDA approval for cosmetic purposes in 2002.^22^ Their findings partially support the present study's finding of dermatologists and plastic surgeons occupying the premium pricing tier and significantly greater odds of access to these practitioners in the least deprived quintile. Regression analysis identified practitioner background as the strongest determinant of pricing, with doctors charging 32% to 38% more than aestheticians, dentists 28% to 33% more, and nurses 2% to 4% more. These relative premiums align with training and regulatory authority and indicate that a third of observed pricing variation can be explained by practitioner profession. However, the present study's findings of higher concentrations of practitioners in more deprived areas demonstrate greater accessibility. Percentage-based price differentials between professions were far greater than those attributable to geography or premises type, indicating that competition in this market is primarily stratified by practitioner qualification rather than clinic branding or location.

The pattern of abundant access coupled with systematic quality stratification represents a departure from established healthcare economic theory. Hart's inverse care law posits that “the availability of good medical care tends to vary inversely with the need for it in the population served.”^23^ However, Hart's framework was developed for therapeutic healthcare where clinical need drives utilization, whereas aesthetic botulinum toxin represents discretionary treatment, where patients function as consumers making elective purchases rather than patients seeking medical care. Our findings suggest that deregulated markets create abundant supply in deprived areas but with reduced clinical oversight. This occurs because unregulated markets allow nonmedical practitioners to compete directly with medical providers, differentiating through lower pricing, creating market accessibility that traditional regulated healthcare seldom achieves. However, the increased accessibility comes with potential safety trade-offs, as demonstrated by the concentration of beauty salon-based treatments in moderately deprived areas (OR = 2.18) where emergency response capabilities and infection control standards may be inadequate for prescription medication administration.

The magnitude of potential safety implications becomes apparent when considering the scale of this market. With an estimated 900,000 botulinum toxin treatments annually and a previously reported 16% complication rate, ∼144,000 complications could occur each year; however, there is a spectrum of severity, with only the most severe requiring hospital care.^4^ Complications range from transient and mild to severe and debilitating and include physical, mental, and financial.^24^ If even a small fraction of these complications requires NHS emergency intervention, the economic burden becomes substantial. The recent UKHSA botulism alert following adverse reactions to aesthetic procedures provides evidence that these risks are materializing, with unlicensed products being implicated in hospitalizations across the NHS.^3^ These events illustrate that complications arising in the private aesthetic sector can create downstream demands on NHS services.

The socioeconomic implications extend beyond simple access patterns to encompass fundamental questions of health equity. Information asymmetry represents a core driver of healthcare market failure, where patients cannot adequately assess quality differences.^25^ In aesthetic medicine, this asymmetry becomes particularly pronounced when prescription-only medications are administered by nonmedical practitioners in nonclinical settings. Patients cannot, for example, distinguish between the safety profiles of a £187.55 ($240.06 USD) botulinum toxin injection from a doctor vs a £148.44 ($189.00 USD) treatment from an aesthetician, yet the 26% price premium suggests quality differences that may translate into differential risk profiles. The slight concentration of nonmedical practitioners in deprived areas (42.0% vs 38.2% in affluent areas) suggests that information disadvantages might compound with economic constraints, potentially exposing vulnerable populations to systematically higher procedural risks.

The proposed local authority licensing scheme faces complex challenges revealed by our analysis. The public consultation outlined by the UK government seeks to classify botulinum toxin under the “Amber” category, where licensed practitioners “must have relevant oversight by a named regulated healthcare professional (who has gained an accredited qualification to prescribe, administer, and supervise aesthetic procedures),” which will be challenging to implement given nearly a quarter of practitioners in the UK have no healthcare background whatsoever.^26^ Simple restrictions on practitioner types could eliminate accessible but unqualified providers from deprived areas. Maintaining the status quo might perpetuate systematic quality stratification with demonstrated public health risks. Any future regulatory framework will therefore need to balance wide accessibility with consistent safety standards across practitioner and premises types. This requires risk-stratified approaches focusing on treatment settings, training standards, and safety infrastructure.

There are limitations to consider when interpreting these findings. The study design is observational; therefore, causality cannot be inferred from the described associations. The 52% availability of pricing data may introduce selection bias and reporting bias toward practitioners willing to advertise prices publicly, potentially underrepresenting certain practitioner types or geographic areas. The study relies on provider definitions for treatment “areas” which may be subject to misclassification. Our cross-sectional design captures market structure during ongoing regulatory discussions that may have influenced practitioner behavior and market positioning which may change behaviors from normal conditions. A proportion of practitioners who do not advertise online or on social media would not have been captured in this analysis. The true magnitude of digital visibility bias cannot be quantified with available methods and represents an inherent limitation of any online-based mapping study. In addition, we do not have data for the volume of procedures performed by practitioners, and the absence of volume data prevents any meaningful inference about practitioner experience, which may be important for readers interpreting the price gradients. Geographic analysis using postcode-level deprivation indices may not perfectly reflect individual patient socioeconomic status, because some patients may travel considerable distances for treatments. There is also a lack of comparative outcome data examining complication rates, treatment effectiveness, and patient satisfaction across practitioner types and treatment settings, which are important for quantifying whether observed quality stratification translates into meaningful safety differences. The mention of potential differences in safety profile and risk stratification resulting from reduced clinical oversight or nonclinical premises are not measured by the findings of the current study but are representative of broader concerns raised over the unlicensed practice of cosmetic procedures in nonclinical settings.^27-30^

## CONCLUSIONS

This study provides the most comprehensive overview to date of aesthetic botulinum toxin practice in the UK, identifying 19,701 practitioners across 5589 clinics, a more than 4-fold increase compared with 2023 estimates. Practitioner composition has shifted, with growth of nonmedical aestheticians alongside persistent socioeconomic differences in access and premises type. Regression analysis confirmed profession as the strongest determinant of pricing, with doctors commanding substantial premiums over nonmedical providers. Geographic analysis further revealed higher practitioner density but reduced access to specialist oversight in more deprived areas.

These findings demonstrate that the UK aesthetic medicine industry has outpaced regulation, creating a trade-off between accessibility and safety oversight, a dynamic that forthcoming reforms must address directly.

## Supplemental Material

This article contains supplemental material located online at https://doi.org/10.1093/asjof/ojag006.

## Supplementary Material

ojag006_Supplementary_Data

## References

[1] GOV.UK . Consultation launched into unregulated cosmetic procedures. Accessed August 15, 2025. https://www.gov.uk/government/news/consultation-launched-into-unregulated-cosmetic-procedures

[2] GOV.UK . Review of the Regulation of Cosmetic Interventions. Accessed November 19, 2023. https://www.gov.uk/government/publications/review-of-the-regulation-of-cosmetic-interventions

[3] GOV.UK . UKHSA issues warning over botulism. Accessed August 15, 2025. https://www.gov.uk/government/news/ukhsa-issues-warning-over-botulism

[4] Zargaran D, Zoller F, Zargaran A, et al Complications of cosmetic botulinum toxin A injections to the upper face: a systematic review and meta-analysis. Aesthet Surg J. 2022;42:NP327–NP336. doi: 10.1093/asj/sjac036 35178552 PMC9005453

[5] Zargaran D, Zoller FE, Zargaran A, Mosahebi A. Complications of facial cosmetic botulinum toxin A injection: analysis of the UK Medicines & Healthcare Products Regulatory Agency registry and literature review. J Plast Reconstr Aesthet Surg. 2022;75:392–401. doi: 10.1016/j.bjps.2021.05.074 34456155

[6] Mahase E . UK government promises crackdown on “cowboy cosmetic procedures” amid rise in rogue providers. BMJ. 2025;390:r1668. doi: 10.1136/bmj.r1668 40774721

[7] GOV.UK . Licensing of non-surgical cosmetic procedures. Accessed August 15, 2025. https://www.gov.uk/government/consultations/licensing-of-non-surgical-cosmetic-procedures

[8] Zargaran D, Zargaran A, Terranova T, et al Profiling UK injectable aesthetic practitioners: a national cohort analysis. J Plast Reconstr Aesthet Surg. 2023;86:150–154. doi: 10.1016/j.bjps.2023.06.057 37717299

[9] Zargaran D, Zargaran A, Sousi S, et al Cosmetic business mechanics in London: a cross-sectional analysis and audit of ASA compliance. J Cosmet Dermatol. 2023;22:2520–2527. doi: 10.1111/jocd.15750 37017936

[10] Hopkins ZH, Moreno C, Secrest AM. Influence of social media on cosmetic procedure interest. J Clin Aesthet Dermatol. 2020;13:28. PMC7028372 32082468

[11] Montemurro P, Porcnik A, Hedén P, Otte M. The influence of social media and easily accessible online information on the aesthetic plastic surgery practice: literature review and our own experience. Aesthetic Plast Surg. 2015;39:270–277. doi: 10.1007/s00266-015-0454-3 25697277

[12] Geographic Data Service . Index of Multiple Deprivation (IMD)—Dataset. Accessed August 15, 2025. https://data.geods.ac.uk/dataset/index-of-multiple-deprivation-imd

[13] gov.scot . Scottish Index of Multiple Deprivation 2020. Accessed August 15, 2025. https://www.gov.scot/collections/scottish-index-of-multiple-deprivation-2020/

[14] StatsWales . 2019. Welsh Index of Multiple Deprivation [Internet]. Accessed August 15, 2025. https://statswales.gov.wales/Catalogue/Community-Safety-and-Social-Inclusion/Welsh-Index-of-Multiple-Deprivation.

[15] Northern Ireland Statistics and Research Agency . Northern Ireland Multiple Deprivation Measure 2017 (NIMDM2017). Accessed August 15, 2025. https://www.nisra.gov.uk/statistics/deprivation/northern-ireland-multiple-deprivation-measure-2017-nimdm2017

[16] Office for National Statistics . Employee earnings in the UK. Accessed August 15, 2025. https://www.ons.gov.uk/employmentandlabourmarket/peopleinwork/earningsandworkinghours/bulletins/annualsurveyofhoursandearnings/2024

[17] Zargaran A, Sousi S, Zargaran D, Mosahebi A. TikTok in plastic surgery: a systematic review of its uses. Aesthet Surg J Open Forum. 2023;5:ojad081. doi: 10.1093/asjof/ojad081 37868688 PMC10588780

[18] Mironica A, Popescu CA, George D, Tegzeșiu AM, Gherman CD. Social media influence on body image and cosmetic surgery considerations: a systematic review. Cureus. 2024;16:e65626. doi: 10.7759/cureus.65626 39205749 PMC11350482

[19] Grand View Horizon . UK Botulinum Toxin Market Size & Outlook, 2030. Accessed August 15, 2025. https://www.grandviewresearch.com/horizon/outlook/botulinum-toxin-market/uk

[20] Kim J, Kang D. Beyond dollars and scars: the influence of wealth inequality on total expenditure on cosmetic procedures in the United States. J Craniofac Surg. 2024;35:899–902. doi: 10.1097/SCS.0000000000010049 38353550

[21] Alsarraf R, Alsarraf NW, Larrabee WF, Johnson CM. Cosmetic surgery procedures as luxury goods: measuring price and demand in facial plastic surgery. Arch Facial Plast Surg. 2002;4:105–110. doi: 10.1001/archfaci.4.2.105 12020205

[22] Satriyasa BK . Botulinum toxin (Botox) A for reducing the appearance of facial wrinkles: a literature review of clinical use and pharmacological aspect. Clin Cosmet Investig Dermatol. 2019;12:223–228. doi: 10.2147/CCID.S202919 PMC6489637 31114283

[23] Tudor Hart J . The inverse care law. Lancet. 1971;297:405–412. doi: 10.1016/S0140-6736(71)92410-X 4100731

[24] Zargaran D, Zargaran A, Sousi S, et al Quantitative and qualitative analysis of individual experiences post botulinum toxin injection—United Kingdom Survey. Skin Health Dis. 2023;3:e265. doi: 10.1002/ski2.265 37799369 PMC10549845

[25] Project MUSE . Arrow and the Information Market Failure in Health Care: The Changing Content and Sources of Health Care Information. Accessed August 19, 2025. https://muse.jhu.edu/pub/4/article/15609/summary 10.1215/03616878-26-5-1031 11765254

[26] GOV.UK . The licensing of non-surgical cosmetic procedures in England: consultation document. Accessed August 20, 2025. https://www.gov.uk/government/consultations/licensing-of-non-surgical-cosmetic-procedures/the-licensing-of-non-surgical-cosmetic-procedures-in-england

[27] Jang HS, Chung KY, Ho Oh B. Complications from cosmetic procedures performed by non-professionals: a case analysis and review of treatments. Korean J Dermatol. 2014;52:222–229.

[28] Holloway V, Manager P, England R. Protecting patients: why tighter regulation is crucial for UK cosmetic surgery. Bull R Coll Surgeons Engl. 2025;107:172–173. doi: 10.1308/rcsbull.2025.69

[29] Mayer JE, Goldberg DJ. Injuries attributable to cosmetic procedures performed by unlicensed individuals in the United States. J Clin Aesthet Dermatol. 2015;8:35. PMC4633211 26557218

[30] Rossi AM, Wilson B, Hibler BP, Drake LA. Nonphysician practice of cosmetic dermatology: a patient and physician perspective of outcomes and adverse events. Dermatol Surg. 2019;45:588–597. doi: 10.1097/DSS.0000000000001829 30946699 PMC6450566

