# Supplementary material for: Mapping the UK Aesthetic Medicine Industry: Practitioner Profiles, Pricing, and Socioeconomic Gradients in Botulinum Toxin Practice
Source: Aesthet Surg J Open Forum. 2026 Feb 11;8:ojag006. doi: 10.1093/asjof/ojag006 (PMC12892226; doi:10.1093/asjof/ojag006)
Supplement: ojag006_Supplementary_Data [file ojag006_supplementary_data.zip › Supplementary Table 1.docx]

| Nation | Number of Practitioners | Median BoNT-A price (£ per area) | Median Filler price (£ per ml) |
| --- | --- | --- | --- |
| England | 15,832 | 169.11 | 260.82 |
| Scotland | 1,854 | 167.30 | 257.86 |
| Wales | 1,117 | 165.58 | 257.89 |
| Northern Ireland | 898 | 170.92 | 261.89 |

Supplementary Table 1: Practitioner Counts and median prices by UK nation
