# Supplementary figures and images for: Mapping the UK Aesthetic Medicine Industry: Practitioner Profiles, Pricing, and Socioeconomic Gradients in Botulinum Toxin Practice
Source: Aesthet Surg J Open Forum. 2026 Feb 11;8:ojag006. doi: 10.1093/asjof/ojag006 (PMC12892226; doi:10.1093/asjof/ojag006)

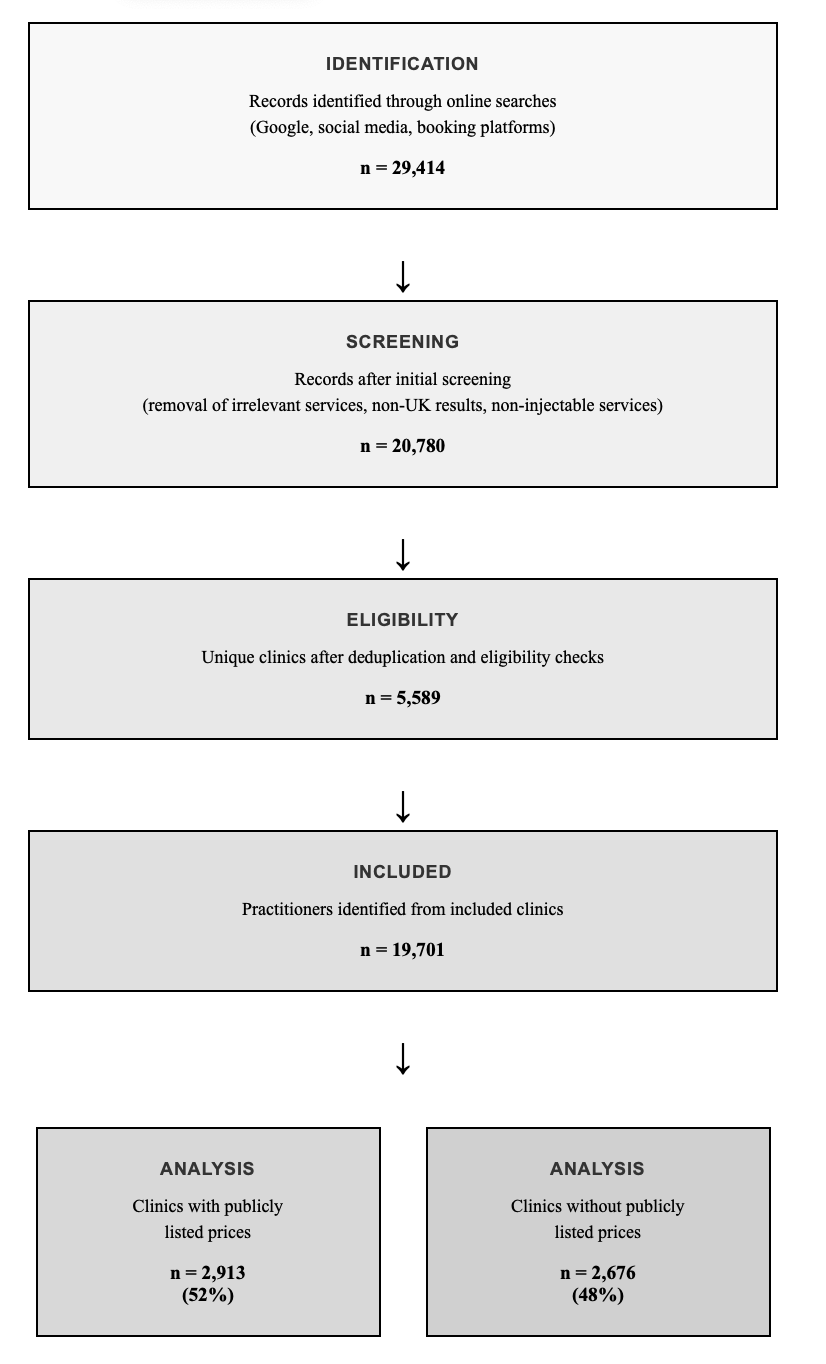

Supplement: ojag006_Supplementary_Data [file ojag006_supplementary_data.zip › Supplementary Figure 1.png]
